# Alzheimer’s disease-associated mutations increase amyloid precursor protein resistance to γ-secretase cleavage and the Aβ42/Aβ40 ratio

**DOI:** 10.1038/celldisc.2016.26

**Published:** 2016-08-23

**Authors:** Ting-Hai Xu, Yan Yan, Yanyong Kang, Yi Jiang, Karsten Melcher, H Eric Xu

**Affiliations:** 1 Key Laboratory of Receptor Research, VARI-SIMM Center, Center for Structure and Function of Drug Targets, Shanghai Institute of Materia Medica, Chinese Academy of Sciences, Shanghai, China; 2 Laboratory of Structural Sciences and Laboratory of Structural Biology and Biochemistry, Van Andel Research Institute, Grand Rapids, MI, USA; 3 University of Chinese Academy of Sciences, Beijing, China

**Keywords:** Alzheimer's disease, C99, γ-secretase, familial Alzheimer disease (FAD)-linked mutations, epsilon-cleavage assay

## Abstract

Mutations in the *amyloid precursor protein* (*APP*) gene and the aberrant cleavage of APP by γ-secretase are associated with Alzheimer’s disease (AD). Here we have developed a simple and sensitive cell-based assay to detect APP cleavage by γ-secretase. Unexpectedly, most familial AD (FAD)-linked APP mutations make APP partially resistant to γ-secretase. Mutations that alter residues N terminal to the γ-secretase cleavage site Aβ42 have subtle effects on cleavage efficiency and cleavage-site selectivity. In contrast, mutations that alter residues C terminal to the Aβ42 site reduce cleavage efficiency and dramatically shift cleavage-site specificity toward the aggregation-prone Aβ42. Moreover, mutations that remove positive charge at residue 53 greatly reduce the APP cleavage by γ-secretase. These results suggest a model of γ-secretase substrate recognition, in which the APP region C terminal to the Aβ42 site and the positively charged residue at position 53 are the primary determinants for substrate binding and cleavage-site selectivity. We further demonstrate that this model can be extended to γ-secretase processing of notch receptors, a family of highly conserved cell-surface signaling proteins.

## Introduction

Alzheimer’s disease (AD) is the most prevalent chronic neurodegenerative disease [1]. It is characterized by the formation of amyloid plaques in the brain that mainly consist of aggregated amyloid beta (Aβ) peptides. Aggregation-prone monomeric Aβ peptides are proteolytic cleavage products of amyloid precursor protein (APP), whose cleavage by β-secretase generates a membrane-bound fragment that contains the C-terminal 99 residues (C99). This fragment is further cleaved by γ-secretase into the intracellular AICD50 peptide and extracellular 37–42 amino-acid amyloid Aβ peptides. Aβ can oligomerize and form higher-order fibrils that give rise to Aβ plaques, whose primary component is the abnormally folded fibrillar form of Aβ [2, 3]. It has also been hypothesized that the aberrant accumulation of the more insoluble and aggregation-prone Aβ42 (42 amino acid Aβ) over Aβ40 is an important trigger for the development and pathogenesis of AD [4]. In an alternative, non-amyloidogenic processing pathway, APP is sequentially cleaved by α secretase to generate a C-terminal 83-residue fragment, and then cleaved by γ-secretase into AICD50 and non-plaque-forming extracellular peptides [5]. Many familial AD (FAD)-linked mutations have been found in the genes encoding γ-secretase and APP, which have led to the hypothesis that pathological processing of C99 and the subsequent formation of Aβ-containing plaques are causally related to AD pathogenesis [6]. Although the molecular basis of AD remains controversial, the amyloid hypothesis, extensively supported by genetics, cell biology, biochemistry and animal studies, has dominated the field of AD research [7–9].

γ-secretase is a multisubunit protease complex that cleaves several type Ι single-pass transmembrane proteins at residues within their transmembrane domains [10]. It is an integral membrane protein, comprising four parts: presenilin (PS), nicastrin, anterior pharynx-defective 1 and presenilin enhancer 2 [11, 12]. PS is the catalytic subunit of γ-secretase [13], whereas the other three subunits are involved in assembly, maturation and stability of the complex [14, 15]. The best-studied substrates of γ-secretase are APP [5, 6] and Notch receptors [16], both of which are of great physiological and pathological importance.

Owing to the central role of γ-secretase in Aβ peptide generation and AD pathology, inhibitors and modulators of γ-secretase have been considered as AD therapeutics, yet little success has been achieved in clinical trials, possibly because of the complexity of substrate recognition and catalytic processing. Although mutations in the gene encoding the catalytic PS component of γ-secretase are strongly associated with early-onset FAD, many disease-associated mutations surprisingly cause a partial loss of function in the γ-secretase complex [17–19]. Although recently the near-atomic structure of γ-secretase has been solved [20–22], the relationship between γ-secretase and its FAD-linked APP substrates remains ambiguous.

**Figure 1:**
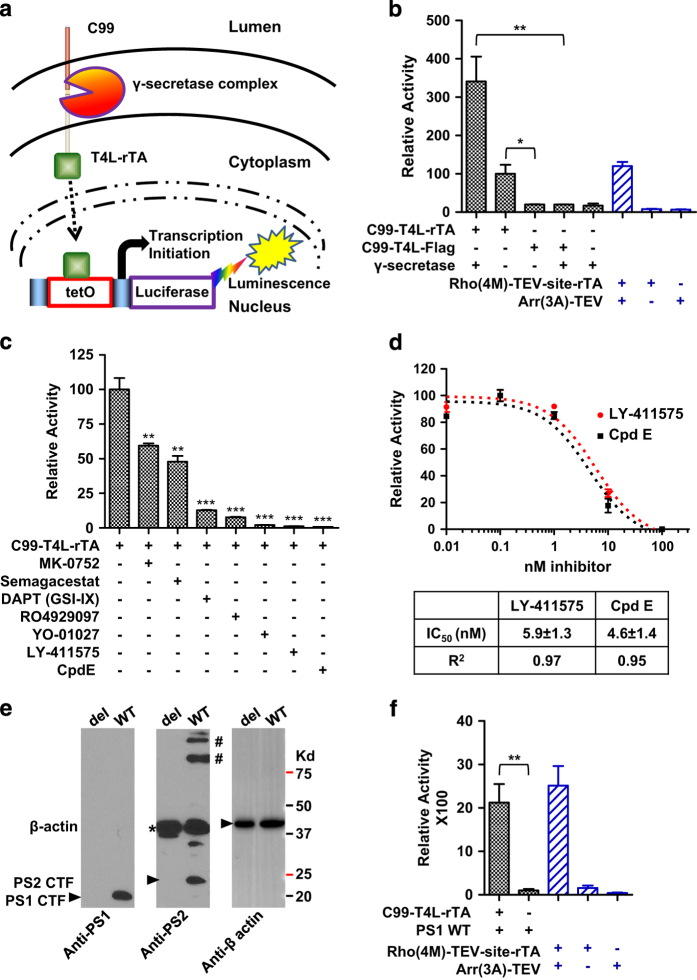
Development and validation of the γ-secretase epsilon-cleavage assay to quantitatively determine relative C99 cleavage by γ-secretase in cells. (**a**) Schematic overview of the γ-secretase epsilon-cleavage assay. Upon membrane cleavage of the C99 hybrid protein by γ-secretase, Aβ peptides are released into the medium and the AICD-T4L-rTA hybrid protein is released from the membrane into the cytoplasm. This allows the hybrid protein to enter the nucleus and to bind tetO DNA-binding site to stimulate luciferase reporter gene activity as measurement for total C99 cleavage, both by endogenous and by transfected γ-secretase variants. (**b**) Relative reporter gene activity using C99-T4L-rTA. Rho(4M)-TEV-site-rTA and Arr(3A)-TEV serve as positive control using a conventional Tango protein–protein interaction assay. (**c**) Cleavage of the C99 hybrid substrate is affected by γ-secretase inhibitors (100 nm per well), indicating that cleavage in HTL cells is due to endogenous γ-secretase. (**d**) IC_50_ dose–response curves for the two most potent γ-secretase inhibitors (LY-411575 compound and compound E (Cpd E)). (**e**) Immunoblot validation of CRISPR/Cas9-mediated chromosomal PS1 and PS2 deletions. PS1 and PS2 protein levels of wildtype (WT) HTL and double-deletion cells are determined by immunoblotting using antibodies that detect the PS1 C-terminal fragment (CTF) (Cell Signaling Technologies 3622S) and antibodies that detect PS2 CFT (Abcam ab106351). β-actin antibody (Abcam ab6276) is used for normalization. *Antibody cross-reactive band. ^#^PS2 membrane protein oligomers. (**f**) Chromosomal deletion of *PS1* and *PS2* abolishes reporter gene activity. Activity can be restored by transfecting WT *PS1* gene to a similar level as the positive control of Rho(4M)-TEV-site-rTA and Arrestin(3A)-TEV. (error bars=s.e.m., *n*=3, *P*-values (two-tailed Student’s *t*-test versus control (**a**, **f**) or WT (**c**)): *P<0.05; **P<0.01; ***P<0.001).

In this study, we have established a variant of the Tango protein–protein interaction assay [23] as a simple and sensitive tool to investigate the cleavage of C99 by γ-secretase in cells. This assay measures the total amount of C99 cleaved, that is, the epsilon-cleavage of C99 irrespective of the number of secondary cleavage events, and which we have therefore termed ‘epsilon-cleavage’ assay (Figure 1a). We tested the effect of all FAD-linked C99 mutations and found that a significant portion of FAD-linked APP mutant proteins became partially resistant to γ-secretase cleavage. In addition, the APP mutations can be divided into two distinct groups, based on whether the mutations alter residues N-terminal (group 1) or C-terminal (group 2) to the Aβ42 cleavage site. Group 1 mutations have subtle effects on cleavage efficiency and Aβ40/Aβ42 specificity. In contrast, group 2 mutations cause partial resistance to γ-secretase cleavage and dramatically skewed cleavage specificity toward Aβ42 production. Unexpectedly, mutations that change the positively charged property at residue 53 nearly abolish APP cleavage by γ-secretase. On the basis of these data, we propose a substrate and charge-recognition model for γ-secretase and APP, which we have validated by Notch substrate cleavage. These results provide a structure–activity relationship profile of the FAD-linked APP mutations as well as molecular insights into substrate recognition and cleavage of APP and Notch receptors by γ-secretase.

## Results

### The epsilon-cleavage assay allows sensitive detection of Aβ cleavage in cells

To investigate the relationship between γ-secretase and its C99 substrate, we developed the γ-secretase epsilon-cleavage assay, which was derived from the cell-based Tango assay [23] and was similar to the Gal4-UAS or Gal4-VP16 systems used previously for Aβ cleavage [24, 25]. The Tango assay is a protein–protein interaction assay, in which a membrane-bound interaction partner is fused to a synthetic tetracycline-controlled transcriptional activator (tTA, comprising a fusion of the tetracycline repressor-TetR with a tandem repeat of the C-terminal activation domain of herpes simplex virus-VP16 [26]) and a TEV protease cleavage site, whereas a soluble interaction partner is fused to TEV protease. Association between the two interaction partners leads to an efficient cleavage of the tTA moiety by TEV protease, which allows tTA to move to the nucleus to activate a luciferase reporter gene. We adopted the Tango assay to test the cleavage of APP by γ-secretase by fusing C99 with a reverse tetracycline-controlled transactivator (rTA), which can mediate tetracycline-independent gene activation upon APP cleavage (Figure 1a). We also introduced T4 lysozyme (T4L) between C99 and rTA to increase substrate stability, and a C-terminal FLAG tag for immunological detection. As a control, we generated the same construct lacking rTA. We then coexpressed these constructs together with the four subunits of human γ-secretase in HEK293-derivative cells with a stably integrated TA-responsive luciferase reporter (HTL cells) [23]. A more than 17× increase in luciferase activity was detected in cells transfected with the C99-T4L-rTA-fused activator or C83-T4L-rTA-fused activator and γ-secretase (Figure 1b and Supplementary Figure S1a). Thus, the Tet-based assays appeared to be more sensitive than the GAL4-based systems [24, 25]. γ-secretase expression was measured by immunoblotting using four antibodies against each subunit of γ-secretase (Supplementary Figure S2a–d).

Compared with a standard Tango assay to detect the interaction between a constitutively active rhodopsin (Rho(4M)) and a pre-activated arrestin (Arr(3A)) [27], the γ-secretase epsilon-cleavage assay showed a similar reporter activity for the C99 fusion construct when combined with the endogenous γ-secretase, but three times higher reporter activity for the C99 fusion construct or C83 fusion construct when co-transfected with γ-secretase (Figure 1b and Supplementary Figure S1a). In contrast, the C99 hybrid lacking rTA failed to elicit reporter gene activity above background (Figure 1b). We further validated the assay by testing the effect of seven different γ-secretase inhibitors [28–32], all of which reduced C99 cleavage to various degrees (Figure 1c). These inhibitors did not affect the reporter activity in the Rho-Arr Tango assay (Supplementary Figure S3), indicating the inhibitor specificity. Inhibition was dose-dependent, as shown in Figure 1d for the two most potent inhibitors, LY-411575 and compound E, as expected for specific inhibition. The complete inhibition by these two inhibitors suggests that the luciferase activity can be all attributed to the γ-secretase cleavage activity toward the C99 substrate reporter.

To further demonstrate that the generated signal is indeed due to cleavage by γ-secretase, as well as to abolish background due to cleavage by endogenous γ-secretase, we introduced internal deletions into both chromosomal isoforms of PS genes, *PS1* and *PS2*, in the HTL cell line using the CRISPR/Cas9 method [33, 34] (see Materials and Methods). Successful genomic deletions were confirmed by immunoblotting with antibodies that recognize the mature PS1 C terminus and the PS2 C terminus (Figure 1e), as well as by quantitative reverse transcriptase polymerase chain reaction (qRT-PCR; Supplementary Figure S5a), PCR and sequencing of the reversibly transcribed PS1 and PS2 mRNAs (Supplementary Figure S5b and c). In the absence of co-transfected γ-secretase expression plasmids, reporter gene activity due to C99 or C83 cleavage was abolished in the PS1/PS2-deleted HTL cells, but could be completely restored by co-transfection with wild-type (WT) PS1 (Figure 1f and Supplementary Figure S1b). PS1 protein expression was shown in Supplementary Figure S2e. This indicates that the relatively high reporter gene signal in the absence of exogenous γ-secretase is indeed completely due to C99 cleavage by endogenous γ-secretase, rather than to cleavage by other proteases. Together, these results demonstrate that we have developed a sensitive cell-based assay for an easy and fast measurement of C99 cleavage by endogenous or transfected γ-secretase in cells.

### Most AD pathogenic C99 mutations reduce C99 cleavage by γ-secretase

**Figure 2:**
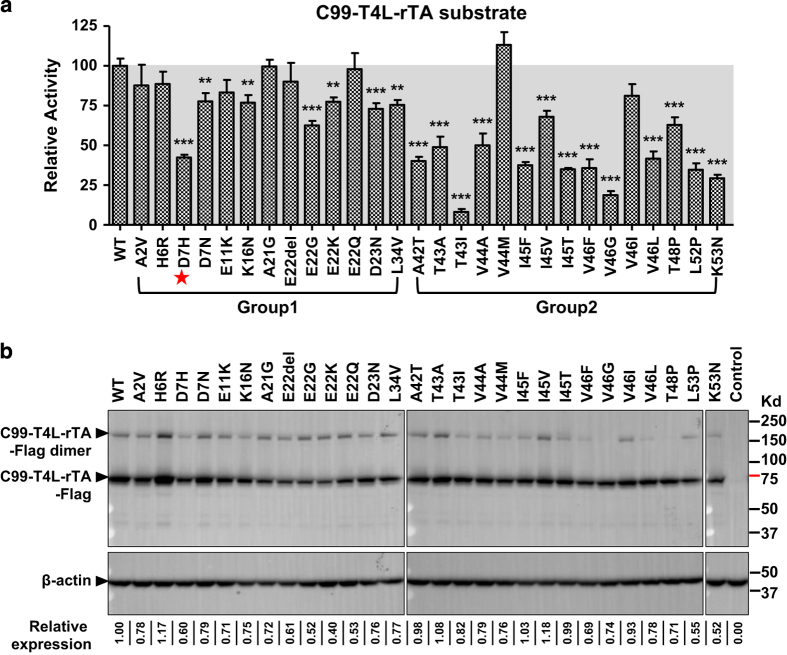
Most FAD-linked C99 mutant proteins are less efficiently cleaved by γ-secretase than WT C99. (**a**) γ-secretase epsilon-cleavage assay in cells expressing WT and mutant C99 hybrid proteins. Most FAD-linked C99 mutations reduce C99-T4L-rTA cleavage by γ-secretase (error bars=s.e.m., *n*=6, *P<0.05; **P<0.01; ***P<0.001 (versus WT)). The red stars indicate the N-terminal key residue in substrate recognition and binding. (**b**) Immunoblot of cells expressing WT and mutant C99-T4L-rTA-Flag proteins, using anti-FLAG antibody for detection and β-actin antibody for normalization. Numbers below the immunoblot provide β-actin normalized signal relative to WT. See Supplementary Figure S9a for normalized activity.

Mutations in the C99 fragment have been associated with clinical onset of AD; however, how these mutations affect their cleavage by γ-secretase has not been systematically examined. To address this question, we introduced all 28 FAD-linked APP single point mutations listed in the Alzforum database (http://www.alzforum.org/mutations, see Supplementary Table S2 for the corresponding C99 positions) into the C99 substrate reporter for the epsilon-cleavage assays. These mutated APP reporters were also introduced to HTL ΔPS1/PS2 cells, in which C99 is not cleaved, for expression-level validation. As shown in Figure 2a, the majority of the pathogenic APP mutations decreased C99 cleavage by γ-secretase, and C99 T43I was almost completely resistant to γ-secretase cleavage. Only 8 of the 28 mutant proteins, the majority of which resided N-terminal to the Aβ42 site (group 1), did not show a statistically significant decrease in reporter gene activity (Figure 2a). Signal decrease was not due to low expression levels as shown by immunoblotting (Figure 2b). The observed decrease in γ-secretase cleavage toward the FAD-linked APP-mutated proteins suggests that these are loss-of-function mutations. The simplest interpretation of these data is that most of the mutant proteins have reduced (or lost) their ability to be efficiently recognized and thus cleaved by γ-secretase. The loss-of-function mutations of FAD-linked APP resemble the scenario of FAD-linked γ-secretase mutations, the majority of which are also loss-of-function mutations [17–19]. Although the β-amyloid hypothesis for AD pathogenesis has paramount supporting evidence, including cellular and animal toxicity of Aβ aggregates, the loss-of-function mutations in FAD-linked APP and γ-secretase remain difficult to be explained and could have important implications in drug discovery for AD. The recent failure of the semagacestat phase III clinical trial adds further complexity for the causal relationship of APP cleavage and γ-secretase activity with AD [35, 36]. The loss-of-function mutations in FAD-linked APP and γ-secretase would suggest that non-selective γ-secretase inhibitors are unlikely to be effective treatments for AD.

### Production of Aβ peptides

**Figure 3:**
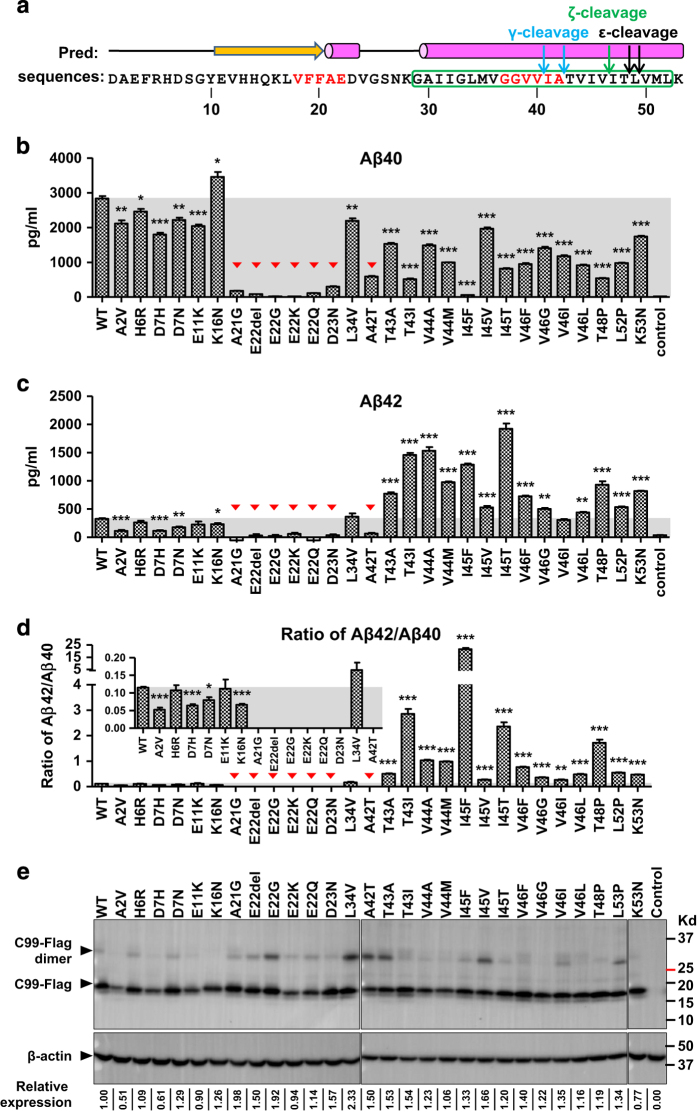
FAD-linked C99 mutations that change amino acids C-terminal to the major γ-secretase cleavage site increase the Aβ42/Aβ40 ratio. (**a**) The sequence of the first 53 residues of C99 with predicted secondary structure elements, γ-secretase cleavage sites and the epitopes (red residues) recognized by AlphaLISA antibodies. (**b**) Concentration of the Aβ40 cleavage product as determined by AlphaLISA. See Supplementary Figure S6 for calibration graph. Mutations (residues A21 to D23 and A42) that affect amino acids within the epitopes recognized by the AlphaLISA detection antibodies are indicated by red triangles. (**c**) Concentration of the Aβ42 cleavage products as determined by AlphaLISA. See Supplementary Figure S6 for calibration graph. (**d**) The ratio of Aβ42 to Aβ40. The inlet shows the ratio at a different scale. The ratios are not presented for mutant proteins in which the mutation site is part of the epitopes. (**e**) Expression levels of C99 mutant proteins determined by anti-FLAG immunoblotting. Numbers below the immunoblot provide β-actin normalized signal relative to WT. Note that because of the sensitivity limitation of the assay (Aβ40: 88 pg ml^−1^; Aβ42: 300 pg ml^−1^), some of the Aβ42 signals were too close to background signal for reliable quantitation. See Supplementary Figure S9b and c for normalized amounts of Aβ40 and Aβ42 (error bars=s.e.m., *n*=3, *P<0.05; **P<0.01; ***P<0.001 (versus WT)).

γ-secretase can cleave C99 at multiple sites, generating intermediate extracellular cleavage products of 43, 45, 46, 48, 49 and 51 amino acids that are further cleaved to the main final products Aβ40 and Aβ42 [37–42] (see Figure 3a). To gain insight into cleavage selectivity, we analyzed the effect of FAD-linked C99 mutants on production of Aβ40 and Aβ42, using a commercial AlphaLISA assay. This assay detects immunologically an epitope that is common to both Aβ40 and Aβ42 (VFFAE) as well as an Aβ42-selective epitope (GGVVIA; see Figure 3a) to determine the levels of Aβ40 and Aβ42, using purified peptides for calibration (Supplementary Figure S6). Wild-type C99 cleavage yielded almost 10 times higher levels of Aβ40 than Aβ42 (Figure 3b versus 3c, Figure 3d inlet), in agreement with the reported ratio [43]. Consistent with the results from the epsilon-cleavage assays (Figure 2), most of the FAD-linked APP mutations reduced Aβ40 cleavage by γ-secretase (Figure 3b). The FAD-linked C99 proteins had similar expression levels (Figure 3e), indicating that differences in Aβ peptide levels are predominantly due to changes in C99 cleavage efficiency by γ-secretase. All mutations altering amino acids N-terminal to the Aβ42 cleavage site were associated with Aβ42 levels close to or below that of the WT APP. In striking contrast, all mutations affecting amino acids in the transmembrane helix C terminal to the Aβ42 cleavage site caused dramatically increased Aβ42/Aβ40 ratios (Figure 3d), mostly because of strong increases in Aβ42 levels together with significant decreases in Aβ40 levels (Figure 3b and c). The dramatic increase in Aβ42 cleavage of V44 and I45 mutations is consistent with the data reported previously [44, 45]. We only found trace levels of Aβ in cell lysates, which were not significantly higher than those of the control cells (Supplementary Figure S7), consistent with the release of Aβ peptides into the extracellular space. Together, these data have important implications in substrate recognition and cleavage of APP by γ-secretase as discussed below.

### Determinants for γ-secretase substrate recognition and cleavage-site selectivity

**Figure 4:**
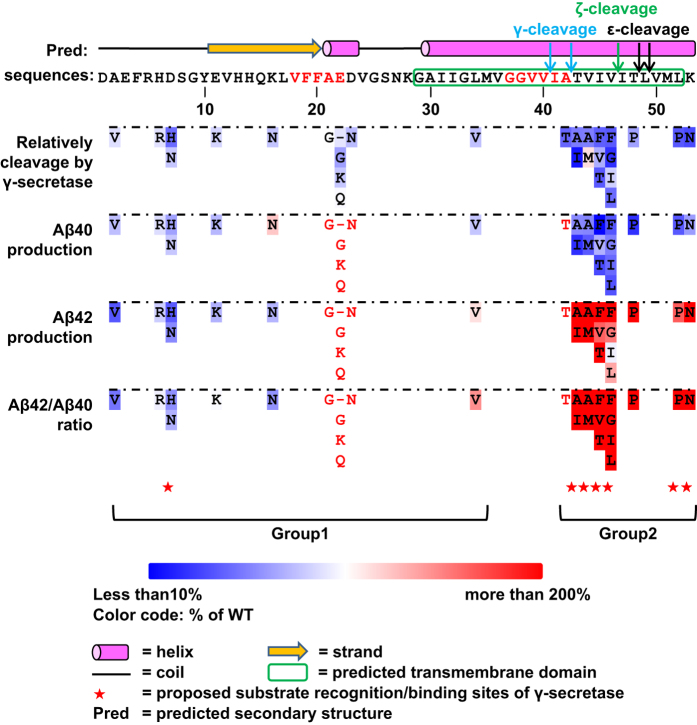
Summary of γ-secretase cleavage efficiency and selectivity for FAD-linked C99 mutant proteins. Top: region of C99 affected by AD-associated mutations together with predicted secondary structure (predicted by PSIPRED Protein Sequence Analysis) [55]. The amino acids of the predicted C99 transmembrane helix are highlighted by a green box, and γ, ϵ and ζ γ-secretase cleavage sites are indicated by arrows. The detection antibody epitopes are indicated by red letters. Middle: amino acids altered by FAD-linked C99 mutations are color-coded based on their effect on total cleavage by γ-secretase and the relative cleavage into Aβ42 and Aβ40 peptides. Red letters indicate mutations in the epitope regions, which are not compared with WT in Aβ40 and β42 production and Aβ42/β40 ratio. Lower panel: color code and symbols for secondary structure elements.

Our results demonstrate that most FAD-linked APP mutant proteins are partially resistant to cleavage by γ-secretase. On the basis of the locations and effects of mutations, the FAD-linked APP mutations can be categorized into two distinct groups. The first group of mutations changes residues N-terminal to the Aβ42 site. Except for mutations in residue D7, mutations in this group show relatively subtle effects on both C99 total cleavage and Aβ42/Aβ40 selectivity and do not exhibit a clear pattern to link these mutations to their processing by γ-secretase (Figures 2 and 3). The second group mutations reside in the C-terminal portion of the Aβ42 (residues 42–53), which appears to be a hotspot of FAD-linked mutations (Figure 4). All FAD-linked mutations in this small region strongly increase the Aβ42/Aβ40 ratio (Figure 3d), with a decrease in the overall cleavage efficiency (Figure 2a) and Aβ40 cleavage (Figure 3b) that is accompanied by an increase in Aβ42 cleavage (Figure 3c).

**Figure 5:**
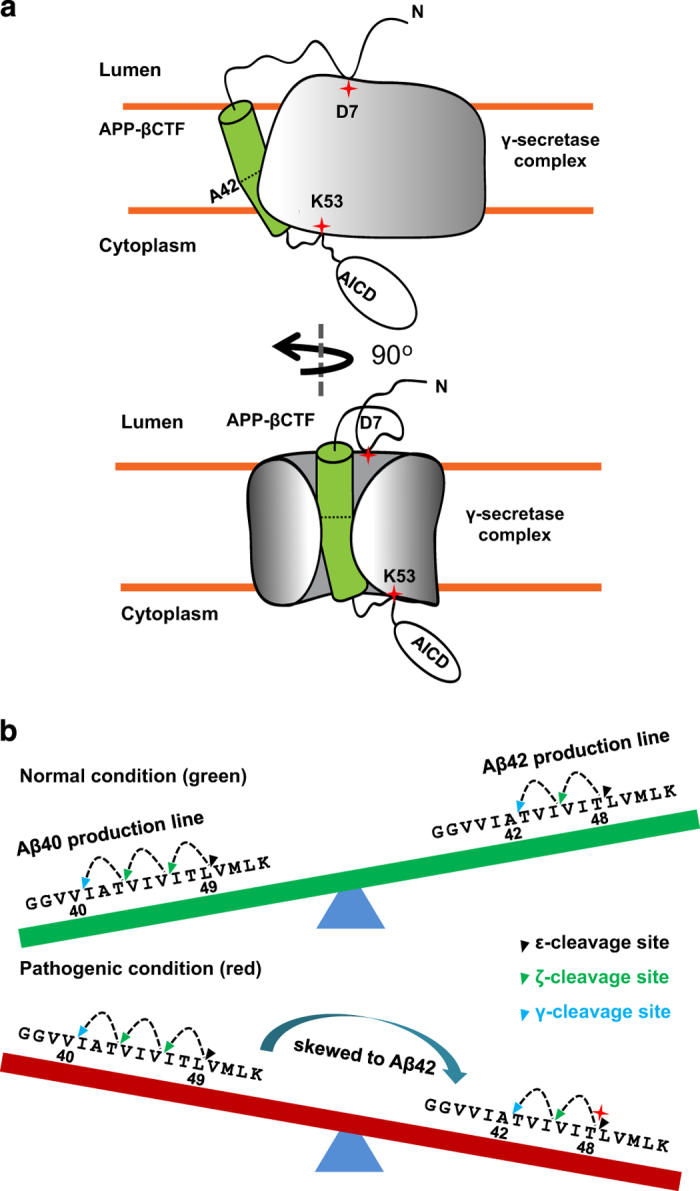
Model of substrate recognition and cleavage-site selection of APP by γ-secretase. (**a**) Schematic diagram of substrate recognition of APP by γ-secretase in two orientations. Red stars indicate the key recognition/binding sites. (**b**) A balance between the two most prevalent Aβ peptide production lines (Aβ40 and Aβ42). Dashed arrowed lines indicate the different γ-secretase cleavage sites with black, green and blue arrows representing ϵ-, ζ- and γ- cleavage, respectively. FAD-linked APP mutations that shift the ϵ-cleavage site from Aβ49 to Aβ48 are marked with a red star.

Currently, there is little information regarding the mechanism of substrate recognition and cleavage-site selectivity of APP by γ-secretase. On the basis of the relation between the sites of FAD-linked APP mutations and γ-secretase cleavage (Figures 2a and 3a), we derive a model of substrate recognition of APP by γ-secretase (Figure 5a). In this model, we propose that the C-terminal region of the APP transmembrane domain (residues 42–53) is the primary substrate recognition/binding site of γ-secretase because most mutations in this region significantly reduce APP cleavage by γ-secretase (group 2 in Figures 2a and 3a). In contrast, mutations in APP residues N-terminal to Aβ 42 have much less effect. Additional substrate recognition and binding may be contributed by residue K53 C-terminal to the APP transmembrane helix, and to a lesser extent by residues D7 from the N terminus of APP because APP mutations in both positions show decreased cleavage efficiency by γ-secretase (Figure 2a).

**Figure 6:**
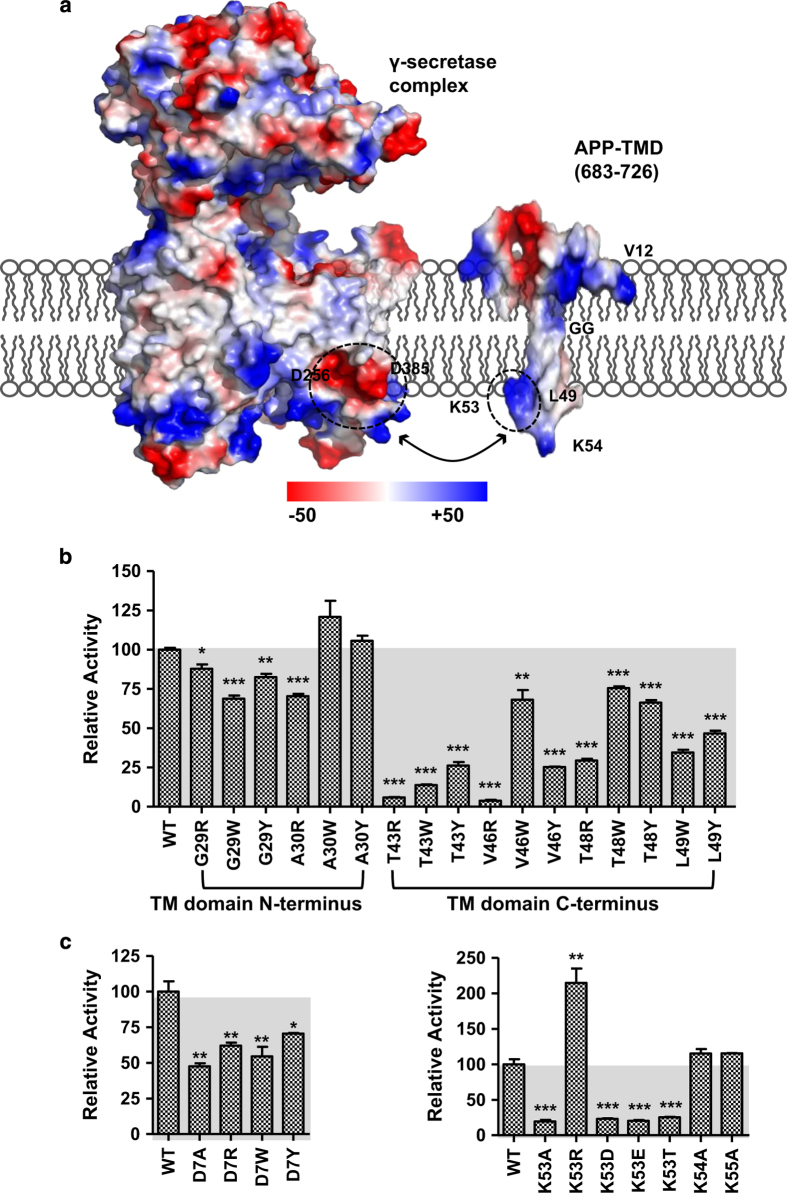
A γ-secretase–APP recognition model and its validation by mutations. (**a**) Charge distribution of human γ-secretase (PDB code: 5A63) and the APP TM domain (PDB code: 2LP1). The two potential charge interaction areas are encircled with dashed lines. (**b**) Epsilon-cleavage assay cleavage efficiency of C99 proteins with mutations within the transmembrane domain (TMD) region indicates that the C-terminal region of TMD is more important than the N terminus of TMD. (**c**) Epsilon-cleavage assay validation of an N-terminal key residue (D7, left panel) and C-terminal key residue (K53, right panel) in C99 cleavage by γ-secretase (error bars=s.e.m., *n*=3, *P<0.05; **P<0.01; ***P<0.001 (versus WT)).

The above model of APP substrate recognition by γ-secretase helps to resolve a key puzzle regarding the cleavage-site selectivity of APP. It has been established that Aβ42 and Aβ40 are generated by sequential tri-peptide cleavage of Aβ48 (or Aβ51) and Aβ49, respectively, which are the initial ϵ-cleavage sites of γ-secretase [42]. However, it remained unclear why mutations C-terminal to the Aβ42 cleavage site affect the cleavage selectivity between Aβ42 and Aβ40 if the mutation site is already removed before the Aβ42/40 cleavage based on the sequential cleavage steps. Our model of substrate recognition indicates that binding of the APP segment C-terminal to Aβ42 determines the initial ϵ-cleavage sites of γ-secretase (Figure 5b). For WT APP, we reason that the default ϵ-cleavage site is preferential for Aβ49 over Aβ48 as the Aβ42/Aβ40 ratio is ~1–10. Mutations that alter the C terminus of APP affect not only substrate-binding efficiency but also the initial cleavage-site selectivity, some of which tip the γ-secretase ϵ-cleavage sites toward Aβ48, which subsequently generates Aβ45 and Aβ42, thus resulting in a higher ratio of Aβ42/Aβ40. This reasoning is consistent with that mutations in the C-terminal APP, including T48P, L52P and K53N (Figure 3), are near the initial γ-secretase ϵ-cleavage sites at Aβ49 or Aβ48. The proposed model of the C-terminal APP amino acids (residues 42–53) as the major determinants for substrate binding and cleavage-site selectivity is consistent with the catalytic site of γ-secretase revealed by the cryo-electron microscopy (cryo-EM) structure [22] (Figure 6a), which is located at the cytoplasmic side that is predicted to be close to the C terminus of the APP transmembrane helix.

There are two predictions from the proposed model of substrate recognition of APP by γ-secretase. The first prediction is that the C-terminal portion of the APP transmembrane helix will be much closer to γ-secretase than the APP N-terminal portion; thus, mutations introducing large side-chain residues at the C-terminal portion of the APP transmembrane domain (TMD) will have more pronounced effects than the mutations in the N terminus of APP TMD. As shown in Figure 6b, large residue mutations at the APP TMD C-terminal portion greatly reduce its cleavage by γ-secretase, whereas corresponding mutations at the APP TMD N-terminal portion have little effect. The second prediction is that mutations in D7 and K53, other than their naturally occurring APP mutations, could also affect γ-secretase cleavage efficiency. This is indeed the case as shown in Figure 6c. D7 mutations (D7A, D7R, D7W and D7Y) show similar effect as the FAD-linked mutations (D7H and D7N). Strikingly, mutations at K53 that remove its positive charge nearly abolish APP cleavage by γ-secretase, whereas K53R mutation does not (which actually increases cleavage), indicating a requirement for a positively charged residue (K53 or R53) for cleavage of APP by γ-secretase (Figure 6c). The effect of K53 mutation is specific as the mutations in the following positively charged residues K54 and K55 have little effect (Figure 6c). The γ-secretase is an aspartic protease with two negatively charged residues at its catalytic site. On the basis of the charge distribution of human γ-secretase (PDB code: 5A63 [22]) and the APP TM domain (PDB code: 2LP1 [46]), we propose a charge-recognition model for γ-secretase substrate recognition, in which the positively charged cluster at K53 would form electrostatic interactions with the negatively charged catalytic pocket (Figure 6a).

### A general model of γ-secretase substrate recognition

**Figure 7:**
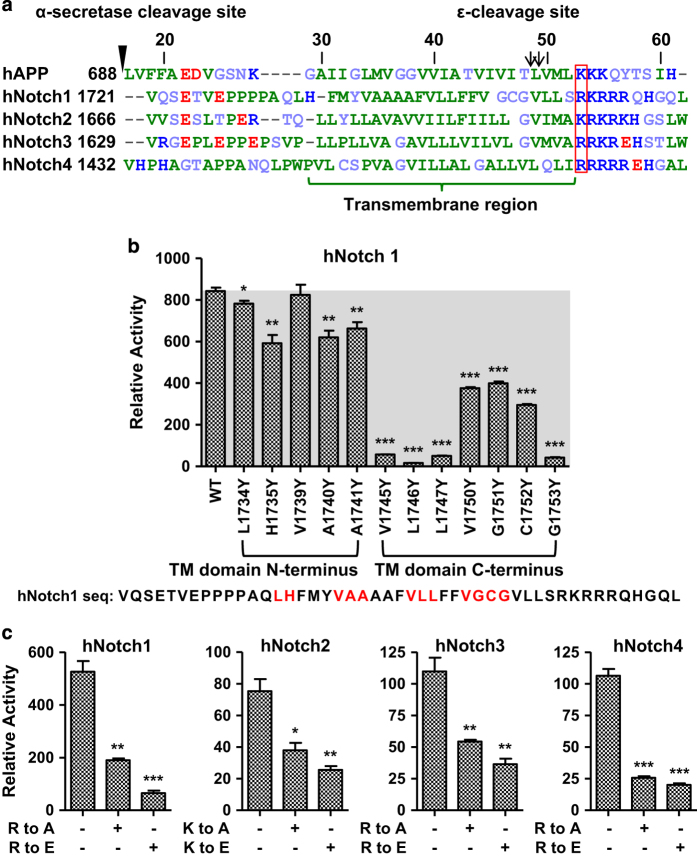
Model extension to Notch substrates. (**a**) Sequence alignment of a subset of known γ-secretase substrates (human APP and human Notch substrates) within the known α-like cleavage site and the 10 N-terminal residues of the intracellular domain (ICD). The column of arginine and lysine residues highlighted in the red box is the conserved positively charged TM junction residue corresponding to K53 in C99. The green bracketed region corresponds to the hydrophobic TM domain. The ϵ-cleavage sites and α-secretase cleavage site are indicated by black arrows and triangle, respectively. Blue: positively charged residues; red: negatively charged residues; green: hydrophobic residues; purple: polar uncharged residues. (**b**) Epsilon-cleavage assay cleavage efficiency of Notch1 proteins with mutations within the TM domain indicate that the C-terminal region of Notch 1 TMD is more important than its N-terminal TMD for cleavage. The corresponding amino acids of Notch1 are shown below the graph. Red letters indicate the mutated residues. (**c**) Replacement of the positively charged TM junction residue in the four human Notch-mini proteins with alanine or glutamic acid strongly reduced notch cleavage (error bars=s.e.m., *n*=3, *P<0.05; **P<0.01; ***P<0.001 (versus WT)).

Besides APP, γ-secretase processes many other substrates, including the family of Notch receptors [47, 48]. We further tested whether our model of APP recognition by γ-secretase can be extended to Notch receptors. First, we introduced human Notch substrates within the known α-like cleavage site and 10 N-terminal residues of the intracellular domain to our system (Figure 7a) and focused on the TM helix of Notch 1. Similar to APP mutations, mutations introducing large residues at the C-terminal portion of the Notch 1 TM helix have much bigger effects than the corresponding mutations at the N-terminal portion (Figure 7b), suggesting that the C-terminal portion of Notch 1 TMD is the primary recognition site of γ-secretase. We then mutated the positively charged residues that follow the Notch TMD to alanine or a negatively charged glutamic acid. In all four Notch receptors, these mutations dramatically decreased γ-secretase cleavage efficiency (Figure 7c), in excellent agreement with the model of APP substrate recognition by γ-secretase. On the basis of sequence alignment of 69 γ-secretase substrates (Supplementary Figure S8), a positively charged residue is 100% conserved at the TM junction (three to four residues after the ϵ-cleavage sites), followed by a cluster of positively charged residues. In the case of N-cadherin, this cluster of positively charged residues is required for its cleavage by γ-secretase [49]. The absolute requirement of the positively charged residues at K53, together with the similarity of the TMD mutation profile between APP and Notch, indicates that the model of APP recognition and cleavage is generally applicable to other γ-secretase substrates, including Notch receptors.

## Discussion

In this study, we have developed a simple cell-based γ-secretase epsilon-cleavage assay to determine the effects of the known FAD-linked APP mutations on C99 cleavage. Together with the measurements of the levels of the most critical cleavage products, Aβ40 and Aβ42, these experiments provide three important observations. First, most FAD-linked APP mutations cause partial resistance to cleavage by γ-secretase (Figure 2a), suggesting that these mutants are less efficiently recognized by γ-secretase. Second, FAD-linked APP mutations can be categorized into two distinct groups based on the locations and effects of mutations. Only mutations that affect residues C-terminal to the Aβ42 cleavage site (residues 42–53, which are a hotspot of FAD-linked mutations; Figure 4) markedly affect cleavage efficiency and strongly increase the Aβ42/Aβ40 ratio. These data suggest that the main γ-secretase recognition site is C-terminal to amino acid 42, and that mutations in this region determine the Aβ42/Aβ40 ratio by skewing the relative efficiencies of the ϵ-cleavage sites at residues 48 or 51 (precursors for Aβ42) and 49 (precursor for Aβ40; Figure 5b). These observations are consistent with previous results for a partial list of FAD-linked APP mutations [50–53]. Third, mutations that remove the positive charge from the invariant lysine or arginine residue at the TM junction (K53 for C99) greatly compromise the cleavage efficiency of APP by γ-secretase. Analysis of the charge distribution of γ-secretase reveals a negatively charged catalytic center that would require a positively charged residue near the initial ϵ-cleavage sites. This conclusion is supported by the conservation of K53 in many γ-secretase substrates (Supplementary Figure S8).

Together, the three key observations above have led us to propose a model of γ-secretase substrate recognition as illustrated in Figure 5a. In this model, the APP region C-terminal to the Aβ42 site serves as the primary site for γ-secretase recognition by being physically closer to γ-secretase than the APP N-terminal region. One possible mechanism for the effect of FAD-linked APP mutations on γ-secretase cleavage is that these APP mutations could cause tilting of the TMD helix, thereby altering the presentation of the substrate to γ-secretase and changing the initial ϵ-cleavage-site selection as proposed previously [53]. Additional determinants for APP recognition by γ-secretase reside in the flanking regions of the APP TM helix, particularly the positively charged residue K53. This model is consistent with all naturally occurring FAD-linked APP mutations (Figures 2 and 3), and we further validate this model extensively with additional mutations that introduce large side-chain residues in the APP TMD helix or mutations that remove the positive charge at residue 53 (Figure 6b and c). A recent study has proposed that the major γ-secretase substrate-binding site resides in the substrate TMD, whereas the ectodomain of substrate is largely dispensable for cleavage [54]. Our model is completely in agreement with this study and further localizes the γ-secretase substrate binding to the TMD region C-terminal to Aβ42 and the conserved positively charged residue at position 53. Importantly, the key features of this model of the APP substrate recognition by γ-secretase are highly conserved in Notch receptors, another major class of γ-secretase substrates (Figure 7). Given the extensive list of γ-secretase substrates, the results and the model presented here should have important implications for how diverse substrates are processed by γ-secretase.

## Materials and Methods

### Cell culture

HTL cells were a gift from G Barnea and R Axel (Brown University and Columbia University). They are derived from HEK293 cells with a stably integrated luciferase reporter under the control of the bacterial tetO operator element. We replaced the tTA with a rTA for increased, tetracycline-independent gene activation [27]. The cells were routinely grown in Dulbecco’s modified Eagle’s medium (Invitrogen Life Technologies, Grand Island, NY, USA) supplemented with 10% (v/v) fetal bovine serum (Invitrogen Life Technologies) at 37 °C under a humidified 5% CO_2_ atmosphere.

### PS1/PS2 deletion cell line

The HTL PS1/PS2-deleted cell line was established by the CRISPR/Cas9 method. In order to express two pairs of single guide RNAs, the expression cassette under the control of the human U6 promoter was repeated four times in the modified pX458 vector (Supplementary Figure S4a). Pairs of single guide RNAs targeting two different sequences within PS1 and PS2, respectively, were designed using the CRISPR Design Tool (http://crispr.mit.edu/; Supplementary Figure S4a and Supplementary Table S1). The modified pX458 vector contains sequences for expression of Cas9, single guide RNAs and green fluorescent protein. Modified pX458 vector (1 μg) was transfected into WT HTL cells using Lipofectamine 2000 (Invitrogen Life Technologies) at a ratio of 2:1 (reagent:DNA) in six-well plates (Millipore, Billerica, MA, USA). After 1-day growth, cells were released with 0.25% trypsin (Invitrogen Life Technologies), pelleted by 3 min centrifugation at 250×g and washed with FACS buffer (2% fetal bovine serum, 2 μM EDTA, 20 mM HEPES in Hank's balanced salt solution (HBSS)). pX458-transfected as well as DAPI-stained cells as live cell control were sorted using flow cytometry, followed by single-cell culture in 96-well plates with 200 μl medium per well (Supplementary Figure S4b). The selected positive cells were validated by western blot and γ-secretase epsilon-cleavage assay (Figure 1e and f and Supplementary Figure S1b) as well as by qRT-PCR, reversibly transcribed mRNA PCR and DNA sequencing (Supplementary Figure S5).

### γ-secretase epsilon-cleavage assay

HTL cells were split at 50 000 per well in a 24-well plate. After 1 day of growth, the cells were transfected with 65 ng total DNA using X-tremeGENE 9 Reagent (Roche Diagnostics, Indianapolis, IN, USA) with a ratio of 1:3 (1 µg DNA:3 µl reagent) according to the standard protocol. For coexpression with γ-secretase, 20 ng substrate-encoding DNA, 5 ng phRG-tk Renilla normalization standard and 10 ng of each of the γ-secretase subunit expression plasmids were transfected. For substrate only, 20 ng substrate, 5 ng phRG-tk Renilla and 40 ng pBSK mock plasmid were transfected with or without proper amount of inhibitors. For the control Tango assay, 10 ng Rho(4M)-TEV-site-rTA, 10 ng Arr(3A)-TEV, 5 ng phRG-tk Renilla and 40 ng pBSK mock plasmid were co-transfected. Cells were harvested and lysed the following day. Luminescence activities were measured using the Dual Luciferase Kit (Promega, Madison, WI, USA) according to the manufacturer’s instructions. Renilla luciferase serves as transfection control. Relative activity is normalized activity using WT C99-T4L-rTA activity as 100.

### RNA isolation and quantitative real-time PCR

Total RNA was extracted from cells with TriZol (Invitrogen Life Technologies), followed by chloroform extraction and isopropanol precipitation. The precipitate was washed with 0.5 ml 75% ethanol, pelleted, air-dried and dissolved in RNase-free water. The SuperScript First-Strand Synthesis Kit (Invitrogen Life Technologies) was used for first-strand complementary DNA synthesis according to the manufacturer’s instructions. Quantitative real-time PCR amplification was carried out in 10 μl solution containing 5 μl Power SYBR Green Real-Time PCR Master Mix (Applied Biosystems, Foster City, CA, USA), 2.5 μl 1:5 diluted complementary DNA sample and 2.5 μl PCR primer mix (forward and reverse each 0.8 μM) using a Step One Plus Thermocycler (Applied Biosystems). Human GAPDH primers (Supplementary Table S1) were used as internal control. The value of threshold cycle (Ct) was generated at every cycle during a run. Fluorescent readings from real-time PCR reactions were quantitatively analyzed by determining the difference of Ct (ΔCt) between the *PS* and *GAPDH* genes. The gene expression of the *PS1* or *PS2* gene was determined as 2^−ΔCt^. The relative gene expression was expressed as percentage of WT control.

### AlphaLISA assay

To get a better profile of Aβ production, we used C99-Flag as substrates and the concentration of transfected DNA was optimized by preliminary tests and set to 320 ng per well with 0.96 µl X-tremeGENE 9 Reagent. After 1 day of growth, the assay was performed as a standard two-step protocol, in which 5 μl cultured supernatant (or cell lysate) were incubated with 5 μl AlphaLISA Aβ1–40/42 acceptor beads and biotinylated anti-Aβ antibody at 23 °C for 1 h, followed by another 30-min incubation with 10 μl AlphaLISA Aβ1–40/42 donor beads in the dark at 23 °C. Photon counts were determined in 384 plates using an Envision-Alpha Reader (PerkinElmer, Waltham, MA, USA).

### Protein isolation and western blot analysis

HTL WT or *PS1*- and *PS2*-deleted cells were transfected with the same amount of DNA as for γ-secretase epsilon-cleavage assays and AlphaLISA assay, respectively, using X-tremeGENE 9 Reagent. For substrate expression quantification, we used HTL *PS1*- and *PS2*-deleted cells. Cells were harvested and lysed in the following day. Western blot analysis was carried out using primary antibodies against FLAG tag (Sigma-Aldrich, St Louis, MO, USA A8592), *PS1* (Cell Signaling Technology, Danvers, MA, USA 3622S), *PS2* (Abcam, Cambridge, MA, USA ab106351), Aph1a (Abcam ab12104), presenilin enhancer 2 (Abcam ab154830), Nicastrin (Abcam ab122969) or β-actin (Abcam ab6276).

### Site-directed mutagenesis

All site-directed mutagenesis was carried out using the QuikChange method (Agilent). All constructs were confirmed using DNA sequencing.

## References

[bib1] Burns A, Iliffe S. Alzheimer's disease. Br Med J 2009; 338: b158. 19196745 10.1136/bmj.b158

[bib2] Zhang YW, Thompson R, Zhang H, Xu H. APP processing in Alzheimer's disease. Mol Brain 2011; 4: 3. 21214928 10.1186/1756-6606-4-3 PMC3022812

[bib3] De-Paula VJ, Radanovic M, Diniz BS, Forlenza OV. Alzheimer's disease. Sub-cell Biochem 2012; 65: 329–352. 10.1007/978-94-007-5416-4_14 23225010

[bib4] Serrano-Pozo A, Frosch MP, Masliah E, Hyman BT. Neuropathological alterations in Alzheimer disease. Cold Spring Harb Perspect Med 2011; 1: a006189. 22229116 10.1101/cshperspect.a006189 PMC3234452

[bib5] Chow VW, Mattson MP, Wong PC, Gleichmann M. An overview of APP processing enzymes and products. Neuromol Med 2010; 12: 1–12. 10.1007/s12017-009-8104-z PMC2889200 20232515

[bib6] Hardy J, Selkoe DJ. The amyloid hypothesis of Alzheimer's disease: progress and problems on the road to therapeutics. Science 2002; 297: 353–356. 12130773 10.1126/science.1072994

[bib7] Pimplikar SW. Reassessing the amyloid cascade hypothesis of Alzheimer's disease. Int J Biochem Cell Biol 2009; 41: 1261–1268. 19124085 10.1016/j.biocel.2008.12.015 PMC2680505

[bib8] Harrington CR. The molecular pathology of Alzheimer's disease. Neuroimaging Clin N Am 2012; 22: 11–22, vii. 22284730 10.1016/j.nic.2011.11.003

[bib9] Selkoe DJ. The molecular pathology of Alzheimer's disease. Neuron 1991; 6: 487–498. 1673054 10.1016/0896-6273(91)90052-2

[bib10] De Strooper B, Vassar R, Golde T. The secretases: enzymes with therapeutic potential in Alzheimer disease. Nat Rev Neurol 2010; 6: 99–107. 20139999 10.1038/nrneurol.2009.218 PMC2879045

[bib11] Fraering PC, Ye W, Strub JM et al. Purification and characterization of the human gamma-secretase complex. Biochemistry 2004; 43: 9774–9789. 15274632 10.1021/bi0494976

[bib12] Kimberly WT, LaVoie MJ, Ostaszewski BL, Ye W, Wolfe MS, Selkoe DJ. Gamma-secretase is a membrane protein complex comprised of presenilin, nicastrin, Aph-1, and Pen-2. Proc Natl Acad Sci USA 2003; 100: 6382–6387. 12740439 10.1073/pnas.1037392100 PMC164455

[bib13] Dang S, Wu S, Wang J et al. Cleavage of amyloid precursor protein by an archaeal presenilin homologue PSH. Proc Natl Acad Sci USA 2015; 112: 3344–3349. 25733893 10.1073/pnas.1502150112 PMC4371958

[bib14] LaVoie MJ, Fraering PC, Ostaszewski BL et al. Assembly of the gamma-secretase complex involves early formation of an intermediate subcomplex of Aph-1 and nicastrin. J Biol Chem 2003; 278: 37213–37222. 12857757 10.1074/jbc.M303941200

[bib15] Li Y, Lu SH, Tsai CJ et al. Structural interactions between inhibitor and substrate docking sites give insight into mechanisms of human PS1 complexes. Structure 2014; 22: 125–135. 24210759 10.1016/j.str.2013.09.018 PMC3887256

[bib16] De Strooper B, Annaert W, Cupers P et al. A presenilin-1-dependent gamma-secretase-like protease mediates release of Notch intracellular domain. Nature 1999; 398: 518–522. 10206645 10.1038/19083

[bib17] Steiner H, Duff K, Capell A et al. A loss of function mutation of presenilin-2 interferes with amyloid beta-peptide production and notch signaling. J Biol Chem 1999; 274: 28669–28673. 10497236 10.1074/jbc.274.40.28669

[bib18] Shen J, Kelleher RJ 3rd. The presenilin hypothesis of Alzheimer's disease: evidence for a loss-of-function pathogenic mechanism. Proc Natl Acad Sci USA 2007; 104: 403–409. 17197420 10.1073/pnas.0608332104 PMC1766397

[bib19] De Strooper B. Loss-of-function presenilin mutations in Alzheimer disease. Talking Point on the role of presenilin mutations in Alzheimer disease. Embo Rep 2007; 8: 141–146. 17268505 10.1038/sj.embor.7400897 PMC1796779

[bib20] Lu P, Bai XC, Ma D et al. Three-dimensional structure of human gamma-secretase. Nature 2014; 512: 166–170. 25043039 10.1038/nature13567 PMC4134323

[bib21] Sun L, Zhao L, Yang G et al. Structural basis of human gamma-secretase assembly. Proc Natl Acad Sci USA 2015; 112: 6003–6008. 25918421 10.1073/pnas.1506242112 PMC4434707

[bib22] Bai XC, Yan C, Yang G et al. An atomic structure of human gamma-secretase. Nature 2015; 525: 212–217. 26280335 10.1038/nature14892 PMC4568306

[bib23] Barnea G, Strapps W, Herrada G et al. The genetic design of signaling cascades to record receptor activation. Proc Natl Acad Sci USA 2008; 105: 64–69. 18165312 10.1073/pnas.0710487105 PMC2224232

[bib24] Zhang C, Khandelwal PJ, Chakraborty R et al. An AICD-based functional screen to identify APP metabolism regulators. Mol Neurodegener 2007; 2: 15. 17718916 10.1186/1750-1326-2-15 PMC2071909

[bib25] Karlstrom H, Bergman A, Lendahl U, Naslund J, Lundkvist J. A sensitive and quantitative assay for measuring cleavage of presenilin substrates. J Biol Chem 2002; 277: 6763–6766. 11744687 10.1074/jbc.C100649200

[bib26] Urlinger S, Baron U, Thellmann M, Hasan MT, Bujard H, Hillen W. Exploring the sequence space for tetracycline-dependent transcriptional activators: novel mutations yield expanded range and sensitivity. Proc Natl Acad Sci USA 2000; 97: 7963–7968. 10859354 10.1073/pnas.130192197 PMC16653

[bib27] Kang Y, Zhou XE, Gao X et al. Crystal structure of rhodopsin bound to arrestin by femtosecond X-ray laser. Nature 2015; 523: 561–567. 26200343 10.1038/nature14656 PMC4521999

[bib28] Wong GT, Manfra D, Poulet FM et al. Chronic treatment with the gamma-secretase inhibitor LY-411,575 inhibits beta-amyloid peptide production and alters lymphopoiesis and intestinal cell differentiation. J Biol Chem 2004; 279: 12876–12882. 14709552 10.1074/jbc.M311652200

[bib29] Dovey HF, John V, Anderson JP et al. Functional gamma-secretase inhibitors reduce beta-amyloid peptide levels in brain. J Neurochem 2001; 76: 173–181. 11145990 10.1046/j.1471-4159.2001.00012.x

[bib30] Mitani Y, Yarimizu J, Saita K et al. Differential effects between gamma-secretase inhibitors and modulators on cognitive function in amyloid precursor protein-transgenic and nontransgenic mice. J Neurosci 2012; 32: 2037–2050. 22323718 10.1523/JNEUROSCI.4264-11.2012 PMC6621706

[bib31] Luistro L, He W, Smith M et al. Preclinical profile of a potent gamma-secretase inhibitor targeting notch signaling with in vivo efficacy and pharmacodynamic properties. Cancer Res 2009; 69: 7672–7680. 19773430 10.1158/0008-5472.CAN-09-1843 PMC5260798

[bib32] Cook JJ, Wildsmith KR, Gilberto DB et al. Acute gamma-secretase inhibition of nonhuman primate CNS shifts amyloid precursor protein (APP) metabolism from amyloid-beta production to alternative APP fragments without amyloid-beta rebound. J Neurosci 2010; 30: 6743–6750. 20463236 10.1523/JNEUROSCI.1381-10.2010 PMC2913973

[bib33] Ran FA, Hsu PD, Wright J, Agarwala V, Scott DA, Zhang F. Genome engineering using the CRISPR-Cas9 system. Nat Protoc 2013; 8: 2281–2308. 24157548 10.1038/nprot.2013.143 PMC3969860

[bib34] Zheng Q, Cai X, Tan MH et al. Precise gene deletion and replacement using the CRISPR/Cas9 system in human cells. Biotechniques 2014; 57: 115–124. 25209046 10.2144/000114196

[bib35] Doody RS, Raman R, Farlow M et al. A phase 3 trial of semagacestat for treatment of Alzheimer's disease. N Engl J Med 2013; 369: 341–350. 23883379 10.1056/NEJMoa1210951

[bib36] Yiannopoulou KG, Papageorgiou SG. Current and future treatments for Alzheimer's disease. Ther Adv Neurol Disord 2013; 6: 19–33. 23277790 10.1177/1756285612461679 PMC3526946

[bib37] Zhao G, Mao G, Tan J et al. Identification of a new presenilin-dependent zeta-cleavage site within the transmembrane domain of amyloid precursor protein. J Biol Chem 2004; 279: 50647–50650. 15485850 10.1074/jbc.C400473200

[bib38] Olsson F, Schmidt S, Althoff V et al. Characterization of intermediate steps in amyloid beta (Abeta) production under near-native conditions. J Biol Chem 2014; 289: 1540–1550. 24225948 10.1074/jbc.M113.498246 PMC3894335

[bib39] Zhao G, Tan J, Mao G, Cui MZ, Xu X. The same gamma-secretase accounts for the multiple intramembrane cleavages of APP. J Neurochem 2007; 100: 1234–1246. 17241131 10.1111/j.1471-4159.2006.04302.x

[bib40] Xu X. Gamma-secretase catalyzes sequential cleavages of the AbetaPP transmembrane domain. J Alzheimers Dis 2009; 16: 211–224. 19221413 10.3233/JAD-2009-0957 PMC2696591

[bib41] Zhao G, Cui MZ, Mao G et al. Gamma-cleavage is dependent on zeta-cleavage during the proteolytic processing of amyloid precursor protein within its transmembrane domain. J Biol Chem 2005; 280: 37689–37697. 16157587 10.1074/jbc.M507993200

[bib42] Takami M, Nagashima Y, Sano Y et al. Gamma-secretase: successive tripeptide and tetrapeptide release from the transmembrane domain of beta-carboxyl terminal fragment. J Neurosci 2009; 29: 13042–13052. 19828817 10.1523/JNEUROSCI.2362-09.2009 PMC6665297

[bib43] Pauwels K, Williams TL, Morris KL et al. Structural basis for increased toxicity of pathological abeta42:abeta40 ratios in Alzheimer disease. J Biol Chem 2012; 287: 5650–5660. 22157754 10.1074/jbc.M111.264473 PMC3285338

[bib44] Suarez-Calvet M, Belbin O, Pera M et al. Autosomal-dominant Alzheimer's disease mutations at the same codon of amyloid precursor protein differentially alter Abeta production. J Neurochem 2014; 128: 330–339. 24117942 10.1111/jnc.12466

[bib45] Chen W, Gamache E, Rosenman DJ et al. Familial Alzheimer's mutations within APPTM increase Abeta42 production by enhancing accessibility of epsilon-cleavage site. Nat Commun 2014; 5: 3037. 24390130 10.1038/ncomms4037 PMC4082030

[bib46] Barrett PJ, Song Y, Van Horn WD et al. The amyloid precursor protein has a flexible transmembrane domain and binds cholesterol. Science 2012; 336: 1168–1171. 22654059 10.1126/science.1219988 PMC3528355

[bib47] Beel AJ, Sanders CR. Substrate specificity of gamma-secretase and other intramembrane proteases. Cell Mol Life Sci 2008; 65: 1311–1334. 18239854 10.1007/s00018-008-7462-2 PMC2569971

[bib48] Haapasalo A, Kovacs DM. The many substrates of presenilin/gamma-secretase. J Alzheimers Dis 2011; 25: 3–28. 21335653 10.3233/JAD-2011-101065 PMC3281584

[bib49] Uemura K, Kihara T, Kuzuya A et al. Characterization of sequential N-cadherin cleavage by ADAM10 and PS1. Neurosci Lett 2006; 402: 278–283. 16687212 10.1016/j.neulet.2006.04.018

[bib50] Hecimovic S, Wang J, Dolios G, Martinez M, Wang R, Goate AM. Mutations in APP have independent effects on Abeta and CTFgamma generation. Neurobiol Dis 2004; 17: 205–218. 15474359 10.1016/j.nbd.2004.04.018

[bib51] Lichtenthaler SF, Wang R, Grimm H, Uljon SN, Masters CL, Beyreuther K. Mechanism of the cleavage specificity of Alzheimer's disease gamma-secretase identified by phenylalanine-scanning mutagenesis of the transmembrane domain of the amyloid precursor protein. Proc Natl Acad Sci USA 1999; 96: 3053–3058. 10077635 10.1073/pnas.96.6.3053 PMC15893

[bib52] Annaert WG, Esselens C, Baert V et al. Interaction with telencephalin and the amyloid precursor protein predicts a ring structure for presenilins. Neuron 2001; 32: 579–589. 11719200 10.1016/s0896-6273(01)00512-8

[bib53] Dimitrov M, Alattia JR, Lemmin T et al. Alzheimer's disease mutations in APP but not gamma-secretase modulators affect epsilon-cleavage-dependent AICD production. Nat Commun 2013; 4: 2246. 23907250 10.1038/ncomms3246

[bib54] Bolduc DM, Montagna DR, Gu Y, Selkoe DJ, Wolfe MS. Nicastrin functions to sterically hinder gamma-secretase-substrate interactions driven by substrate transmembrane domain. Proc Natl Acad Sci USA 2016; 113: E509–E518. 26699478 10.1073/pnas.1512952113 PMC4747693

[bib55] Buchan DW, Minneci F, Nugent TC, Bryson K, Jones DT. Scalable web services for the PSIPRED Protein Analysis Workbench. Nucleic Acids Res 2013; 41: W349–W357. 23748958 10.1093/nar/gkt381 PMC3692098

