# Supplementary material for: Alzheimer’s disease-associated mutations increase amyloid precursor protein resistance to γ-secretase cleavage and the Aβ42/Aβ40 ratio
Source: Cell Discov. 2016 Aug 23;2:16026–. doi: 10.1038/celldisc.2016.26 (PMC4994064; doi:10.1038/celldisc.2016.26)
Supplement: Supplementary Information [file celldisc201626-s1.pdf]

Supplementary information, Figure S1

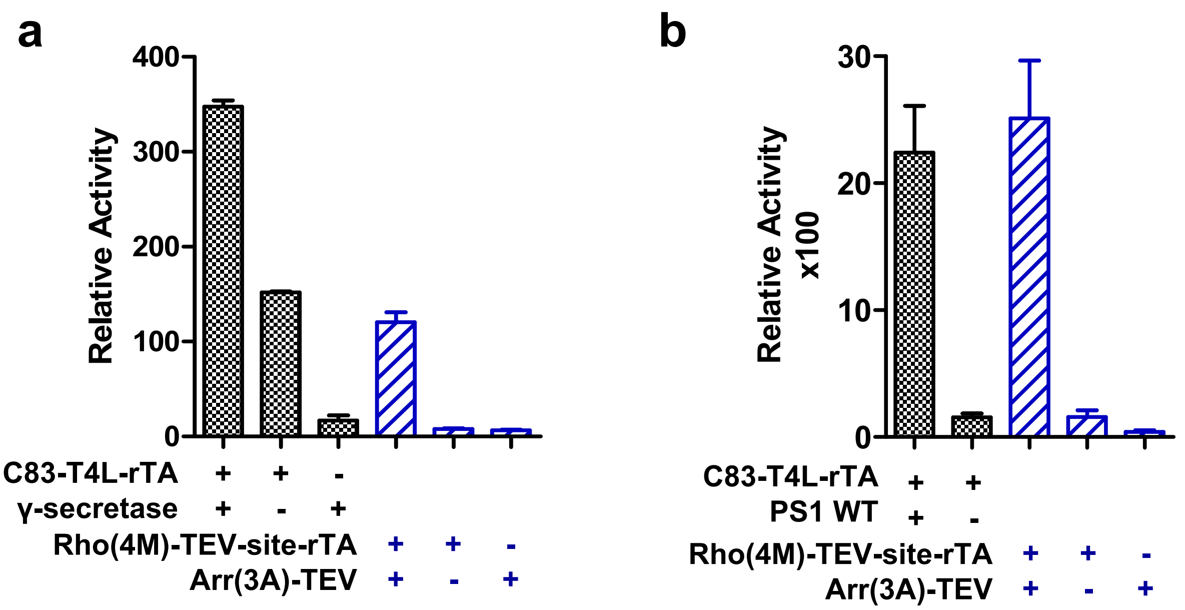

Figure S1: C83 cleavage by γ-secretase (A) Relative reporter gene activity using C83-T4L-rTA as substrate. Rho(4M)-TEV-site-rTA and Arr(3A)-TEV serve as positive control. (B) Reporter gene activity is abolished in cells with a chromosomal deletion of *PS1* and *PS2*. Activity can be restored to a similar level as the positive Rho-Arr control by transfecting wild type *PS1* (first lane).

Supplementary information, Figure S2

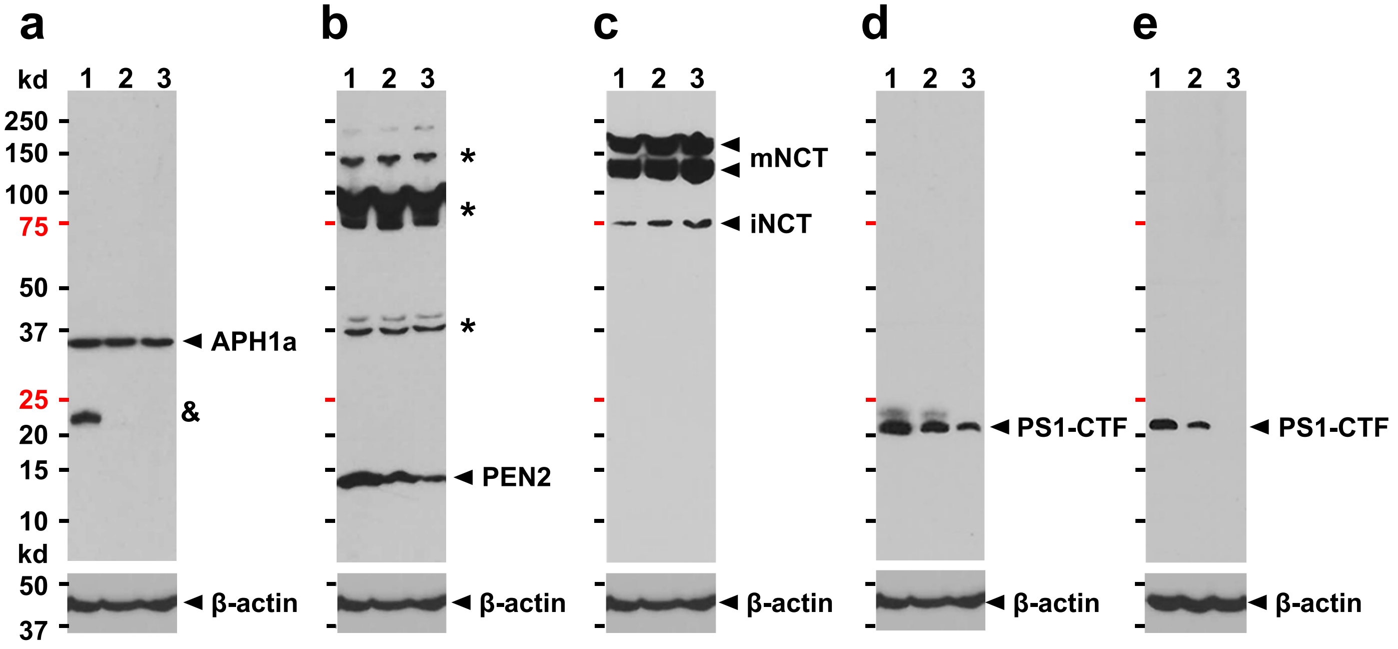

Figure S2: Western blot validation of overexpression of γ-secretase. Lane 1: Extracts from cells co-transfected with the four subunits of γ-secretase; Lane 2: Extracts from cells transfected with PS1 wild type; Lane 3: Extracts from non-transfected cells (endogenous control). In HTL WT cells, γ-secretase protein levels were determined by immunoblotting using Anti-Aph1a antibody (Abcam ab12104) (a), Anti-PEN2 antibody (Abcam ab154830) (b), Anti-Nicastrin antibody (Abcam ab122969) (c), and Anti-PS1 CTF (Cell Signaling Technologies 3622S) (d). In HTL PS deletion cells, PS1 protein levels were determined by immunoblotting using Anti-PS1 CTF (Cell Signaling Technologies 3622S) (e). β-actin antibody (Abcam Ab6276) has been used for normalization (lower panel). [&]APH1a isoform. *Antibody cross-reactive bands.

Supplementary information, Figure S3

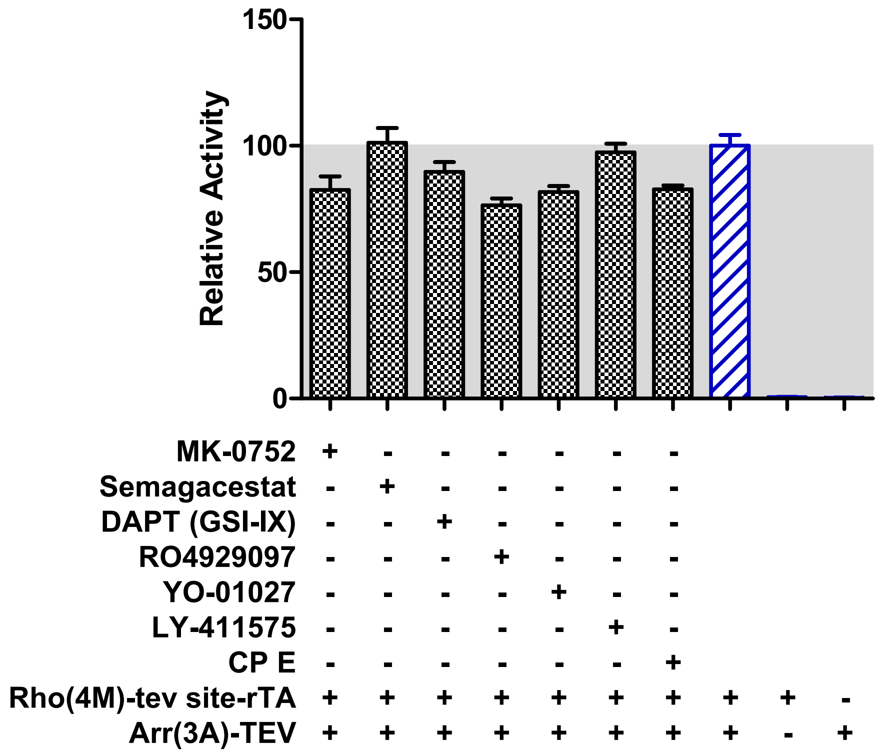

Figure S3: γ-secretase inhibitors do not affect reporter activity in an unrelated Tango assay. All seven inhibitors (100 nM) used in the γ-secretase epsilon-cleavage assay (Figure 1c) have only minor effects on reporter gene activity in the control Rho-Arr Tango assay.

Supplementary information, Figure S4

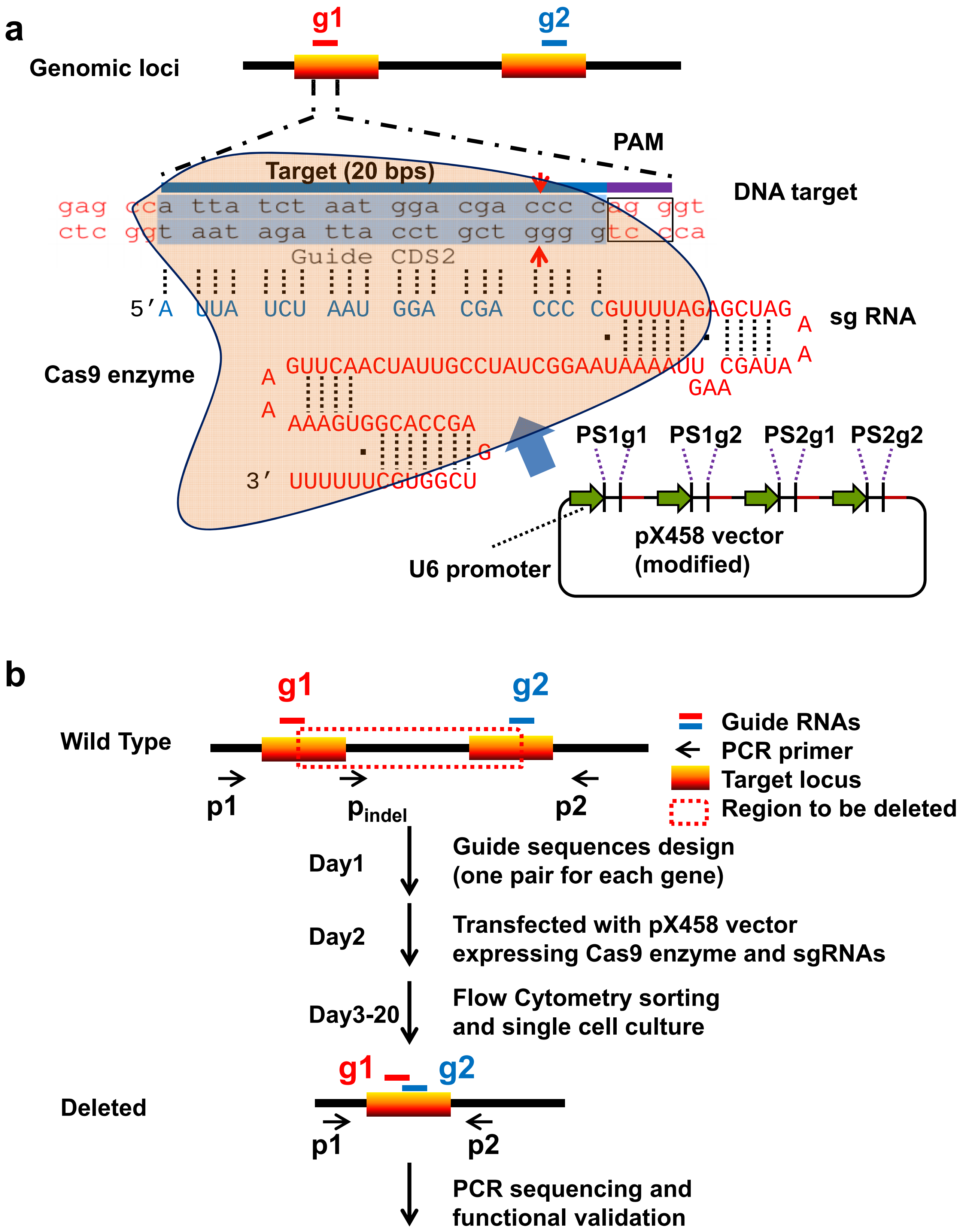

Figure S4: An optimized system for targeted gene deletion using CRISPR/Cas9. (A) Schematic diagram depicting the locations of single guide RNAs targeting human PS gene loci. Guide RNAs were designed using the online CRISPR Design Tool (http://crispr.mit.edu/) and inserted into the modified pX458 vector. Cas9 (light orange shape) guided by sgRNA, which consists of a 20-nt guide sequence (blue) and a scaffold sequence (red), cleaves genomic DNA three base pairs upstream of the requisite 5′-NGG protospacer adjacent motif (PAM (purple); cleavage site indicated by red arrows). The modified pX458 vector is designed to produce one pair of sgRNAs for each gene. (B) Flowchart of targeted gene deletion using CRISPR/Cas9.

Supplementary information, Figure S5

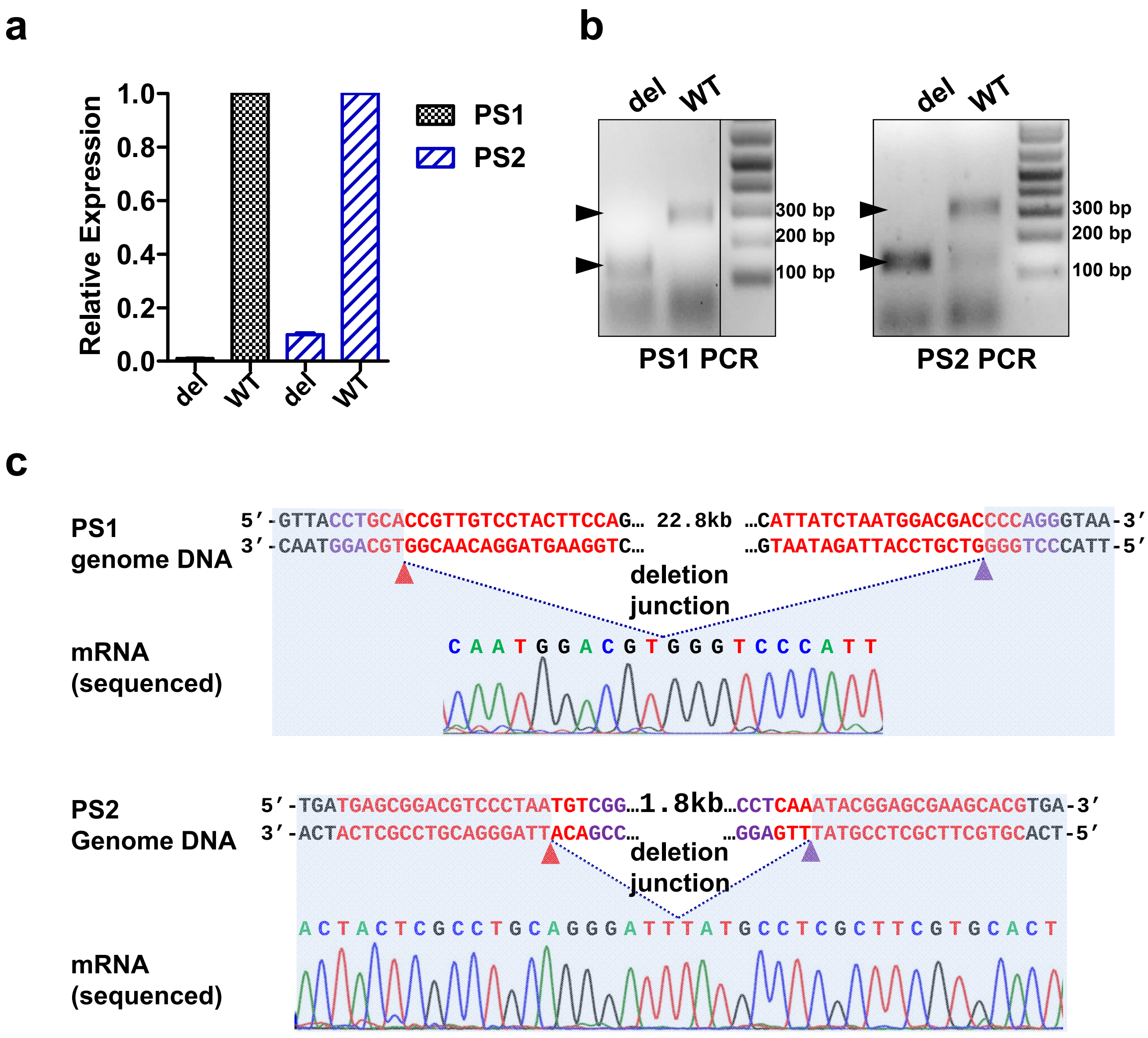

Figure S5: Validation of the PS1/PS2 deletion. (A) Relative expression of PS1 and PS2 mRNA. Quantitate RT-PCR using p_{indel} and P_2 primers (Table S1) and GAPDH as an internal reference (error bars=SEM, n=3). (B) PS1/PS2 cDNA PCR validation (P_1 and P_2 as PCR primers). The fragment for the PS1 deletion has a size of 144 bps while the size of the corresponding WT fragment is 289 bps; the fragment for the PS2 deletion has a size of 134 bps while the one of the corresponding WT fragment is 318 bps. (C) PS1/PS2 cDNA sequencing validation (using P_2 as sequencing primer).

Supplementary information, Figure S6

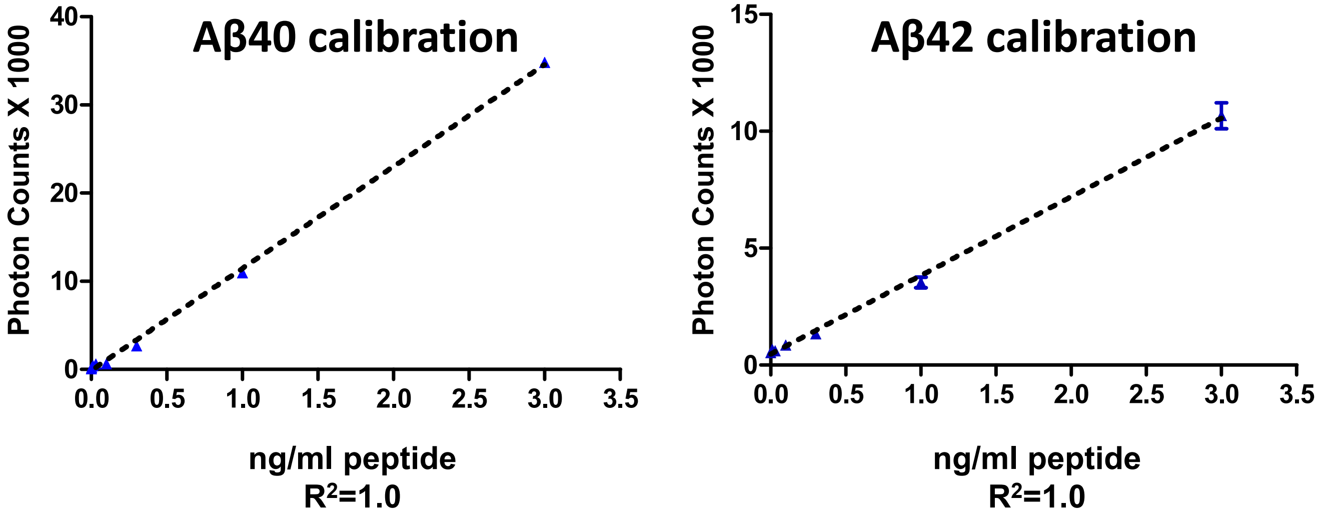

Figure S6: Calibration of Aβ peptide levels. Aβ peptide calibration was carried out according to the PerkinElmer AlphaLISA standard protocol. The sensitivity of the Aβ40 kit (AL202 C/F) is 88 pg/mL and Aβ42 (AL203 C) is 300 pg/mL.

Supplementary information, Figure S7

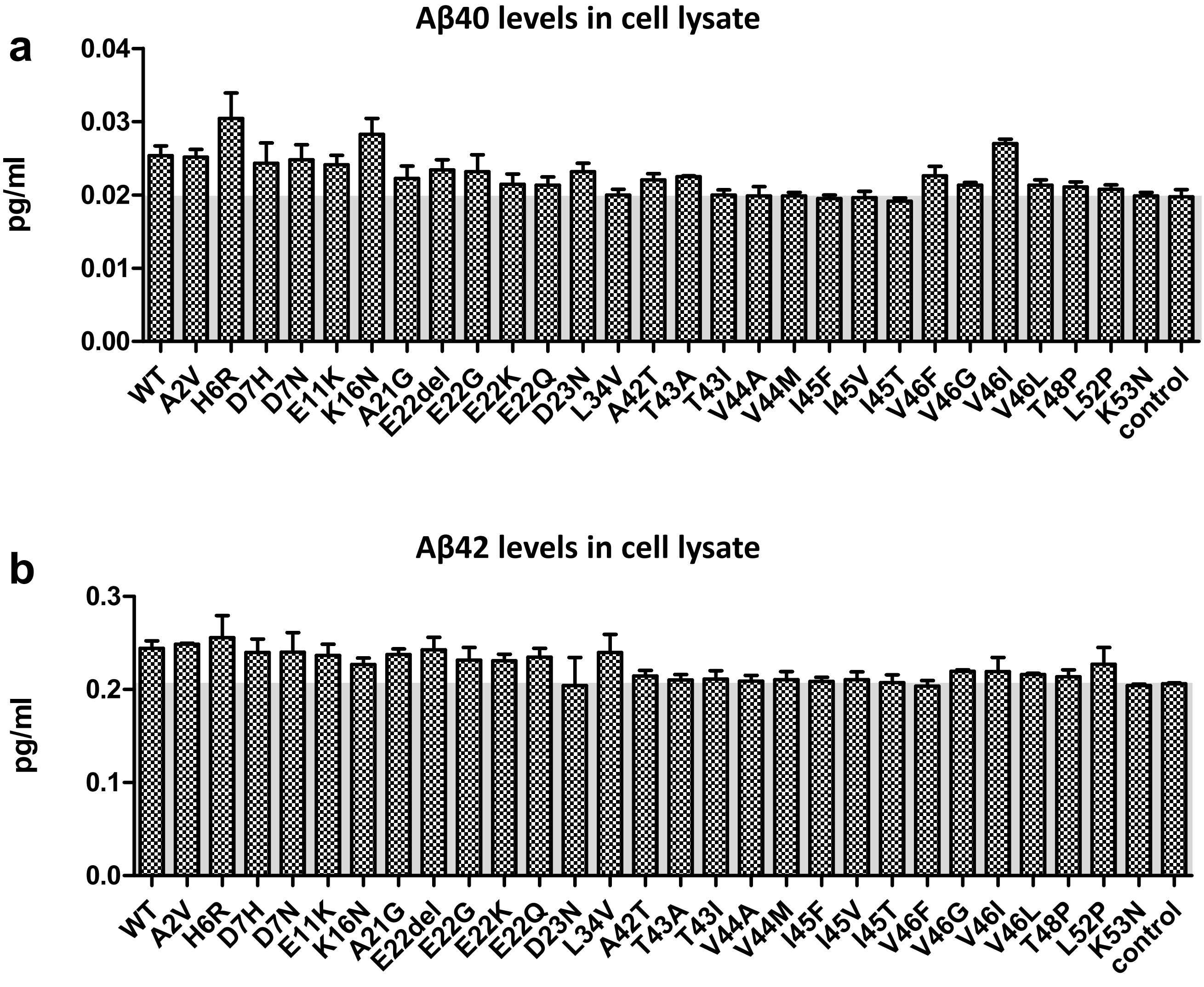

Figure S7: Aβ peptide levels in cell lysate. AlphaLISA assay of lysates from cells transformed with the mutant C99 constructs corresponding to Figure 3. The cell lysate Aβ40 (a) or Aβ42 (b) peptide levels are close to background (control lane). Error bars=SEM, n=3.

Supplementary information, Figure S8

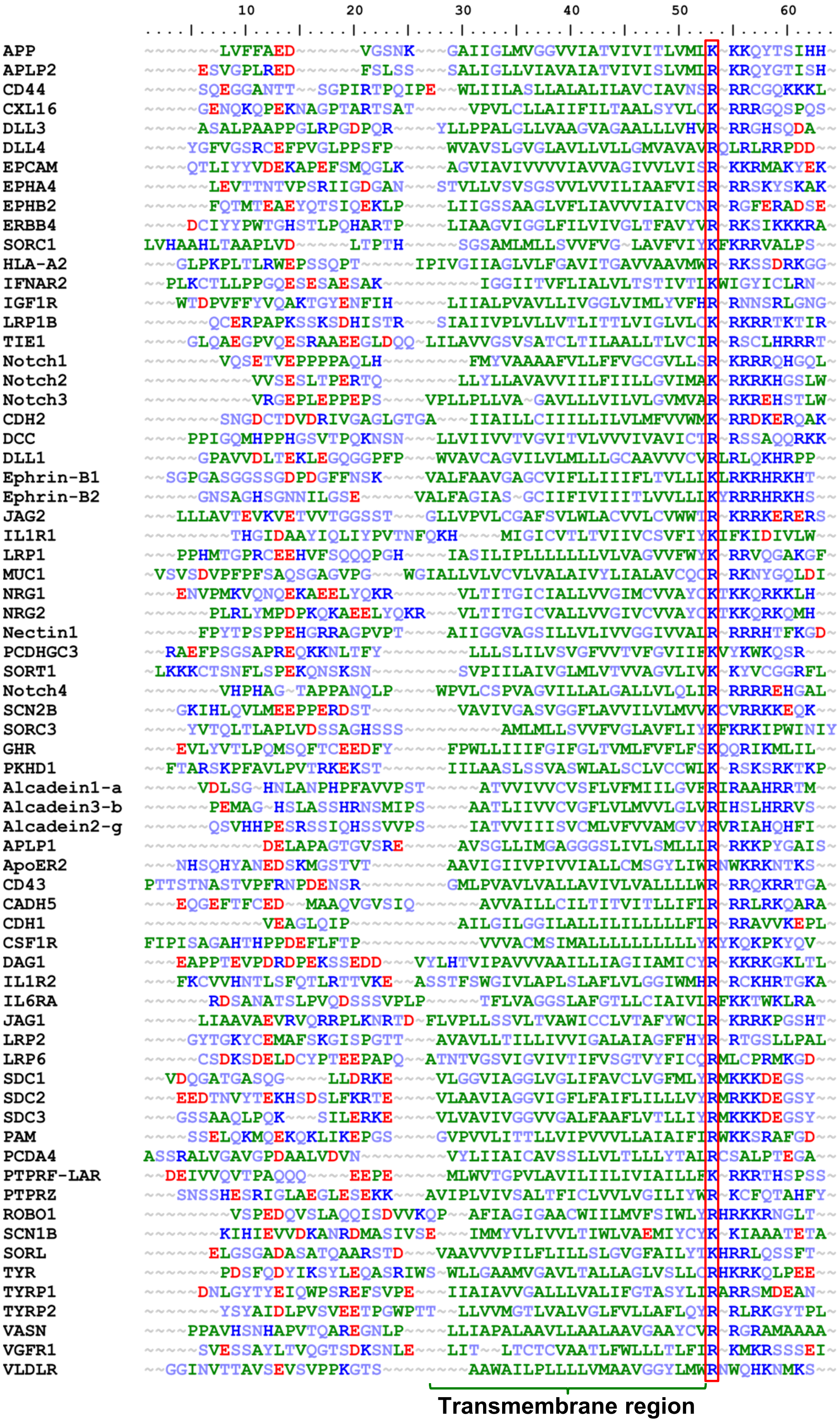

Figure S8: Sequences and properties of γ-secretase substrates. Alignment of the sequences of the 69 γ-secretase substrates with known cleavage sites. Only the last 20 residues of the extracellular domain (ECD; unless cutting at the known site) and the 10 N-terminal residues of the intracellular domain (ICD) are presented. The hydrophobic TM domain is indicated by the green bracket. Boxed arginine and lysine residues are the conserved positively charged residues at the TM junction site. The color code is the same as in Figure 7.

Supplementary information, Figure S9

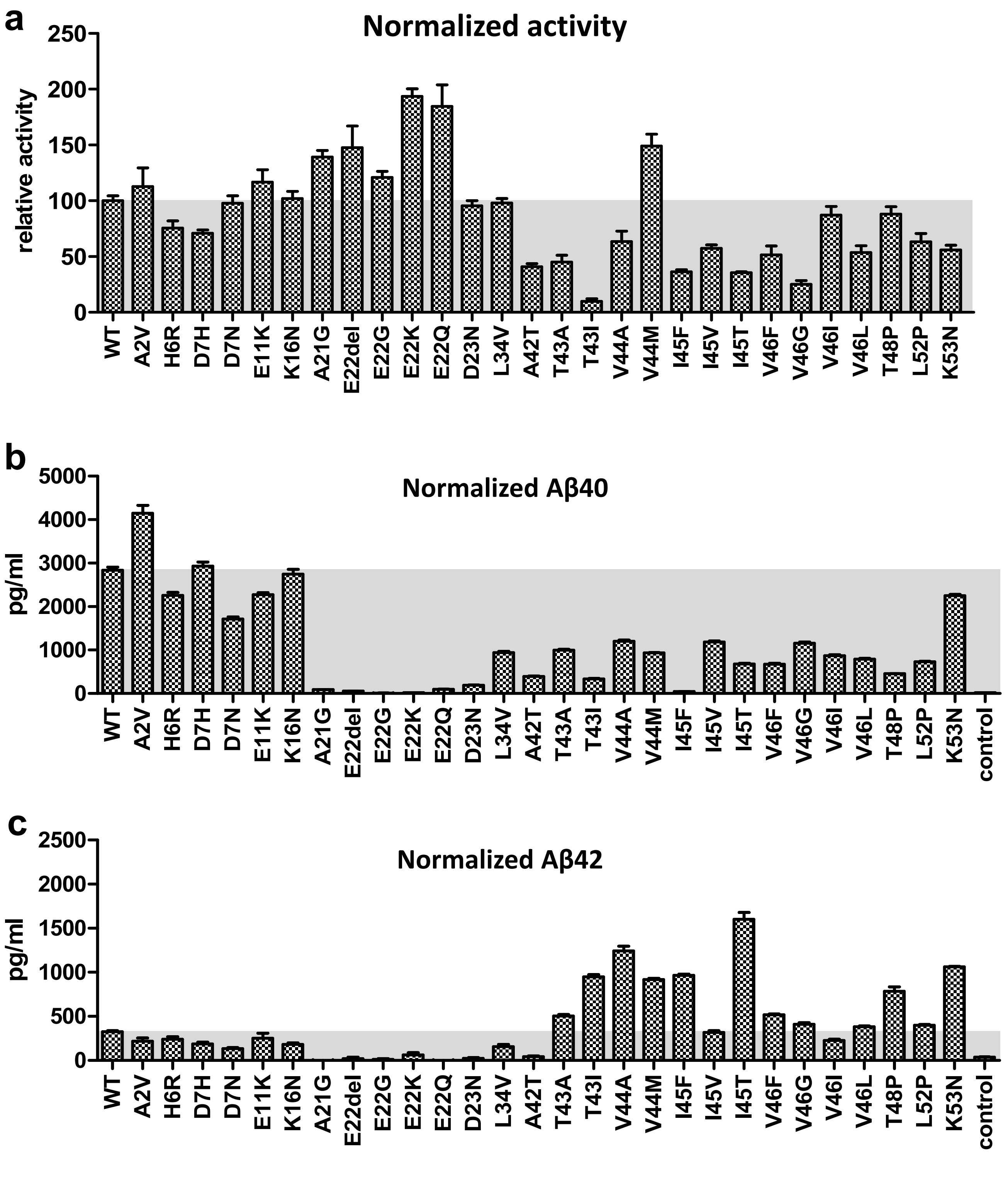

Figure S9: Normalized epsilon-cleavage assay activities (a) and amounts of Aβ40 (b) and Aβ42 (c). Data were normalized to relative protein expression in Figure 2b and Figure 3e, respectively.

Supplementary information, Table S1

Table S1: Oligonucleotide primers used in this study. All primers were synthesized by Integrated DNA Technologies.

Primers used in this study	
name	sequences
PS1-Guide-CDS1-F	CACCGTGGAAGTAGGACAACGGTGC
PS1-Guide-CDS1-R	AAACGCACCGTTGTCCTACTTCCAC
PS1-Guide-CDS2-F	CACCGATTATCTAATGGACGACCCC
PS1-Guide-CDS2-R	AAACGGGGTCGTCCATTAGATAATC
PS2-Guide-CDS1-F	CACCGTGAGCGGACGTCCCTAATGT
PS2-Guide-CDS1-R	AAACACATTAGGGACGTCCGCTCAC
PS2-Guide-CDS2-F	CACCGCGTGCTTCGCTCCGTATTTG
PS2-Guide-CDS2-R	AAACCAAATACGGAGCGAAGCACGC
PS1-P1	TTGCGGTCCTTAGACAGCTT
PS1-Pindel	AATAGAGAACGGCAGGAGCAC
PS1-P2	ACGACCACCACCATGCAGAG
PS2-P1	CTGCCCAGGAGAGAAATGAG
PS2-Pindel	TGACCGCTATGTCTGTAGTGG
PS2-P2	TGTAGAAGCGCACAGACTTG
hGAPDH-614F	TGGAAGGACTCATGACCACA
hGAPDH-776R	TTCAGCTCAGGGATGACCTT

Supplementary information, Table S2

Table S2: FAD-linked C99 mutations. List of FAD-linked APP mutations within C99 as listed in the Alzforum database (http://www.alzforum.org/mutations).

FAD-linked C99 mutations	
**APP Mutations**	**corresponding C99 position**
A673V	2
H677R (English)	6
D678H (Taiwanese)	7
D678N (Tottori)	7
E682K (Leuven)	11
K687N	16
A692G (Flemish)	21
E693del (Osaka, E693∆, E693delta)	22
E693G (Arctic)	22
E693K (Italian)	22
E693Q (Dutch)	22
D694N (Iowa)	23
L705V	34
A713T	42
T714A (Iranian)	43
T714I (Austrian)	43
V715A (German)	44
V715M (French)	44
I716F	45
I716V (Florida)	45
I716T	45
V717F (Indiana)	46
V717G	46
V717I (London)	46
V717L	46
T719P	48
L723P (Australian)	52
K724N (Belgian)	53
